# Supplementary figures and images for: daf-16/FOXO blocks adult cell fate in Caenorhabditis elegans dauer larvae via lin-41/TRIM71
Source: PLoS Genet. 2021 Nov 15;17(11):e1009881. doi: 10.1371/journal.pgen.1009881 (PMC8629381; doi:10.1371/journal.pgen.1009881)

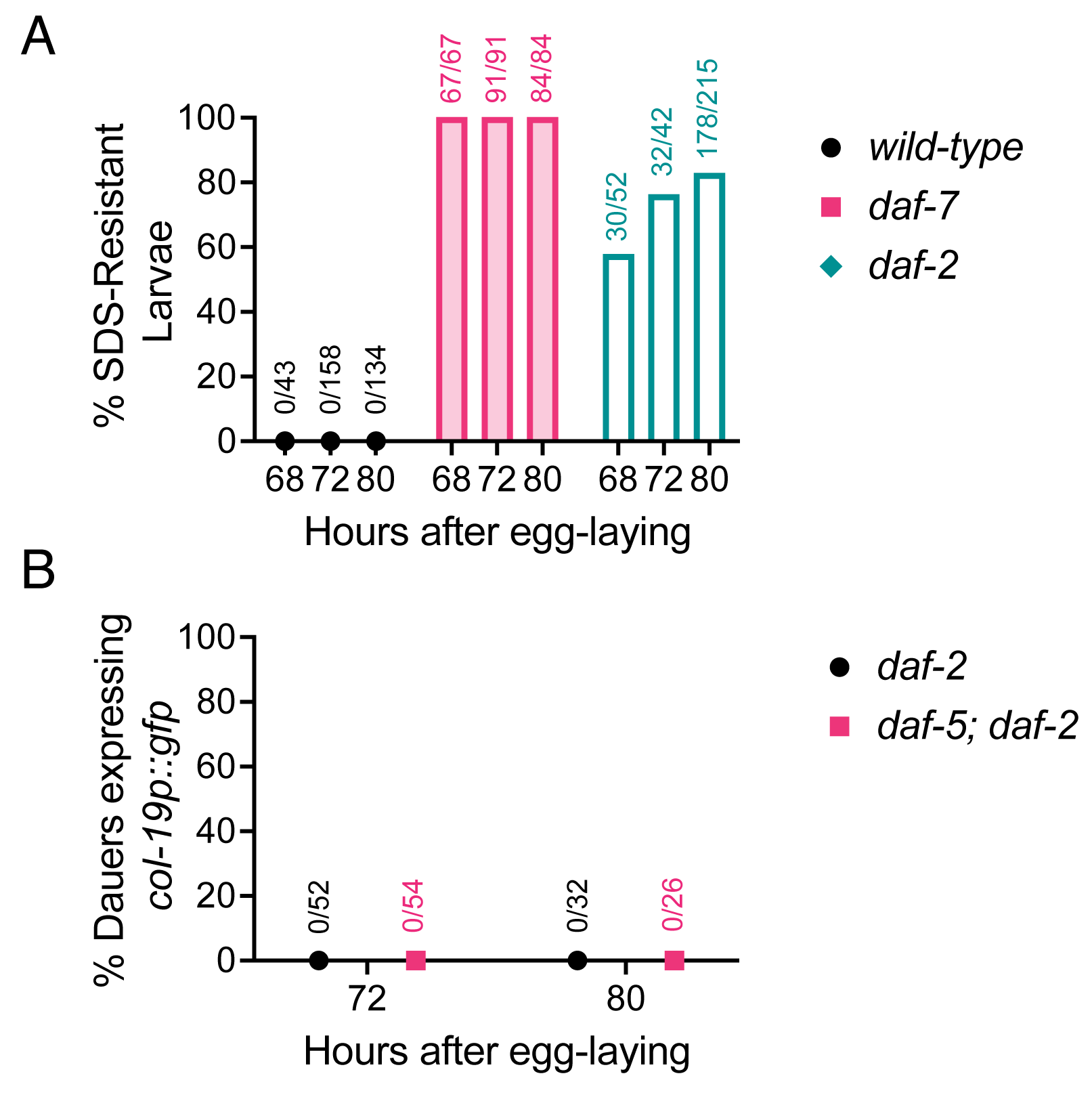

Supplement: S1 Fig — The daf-7/TGFβ pathway regulates dauer formation in parallel to the insulin-like pathway [6,7]. daf-5 encodes a Sno/Ski protein that works in a complex with DAF-3/SMAD to regulate transcription downstream of DAF-7/TGFβ signaling [76]. daf-5(0) mutants are dauer-defective, but can be induced to enter dauer when combined with the daf-2(e1370) allele [34,35]. (A) daf-2(e1370) mutants develop slowly and acquire dauer characteristics approximately one day later than wild-type or daf-7(e1372) larvae [60,77]. Since daf-16; daf-7 dauer larvae express col-19p::gfp most penetrantly within one day of dauer entry, we used SDS-resistance to determine a time that might correspond to “early dauer” in this strain. SDS-resistance is acquired at the end of the L2d-to-dauer molt and continues throughout dauer [2]. Gravid adult hermaphrodites of the indicated strains were allowed to lay eggs for 3 hours and then removed. Embryos were incubated at 24°C for the indicated times and then incubated in 1% SDS for 10 minutes. The percent of larvae alive after this treatment is shown; data were aggregated from four independent experiments. (B) Percent of dauer larvae expressing col-19p::gfp. This expression was never observed in either control or daf-5(0) dauer larvae at times when many early dauer larvae should have been present. Data shown were aggregated from 3–4 independent experiments. (TIF) [file pgen.1009881.s001.tif]

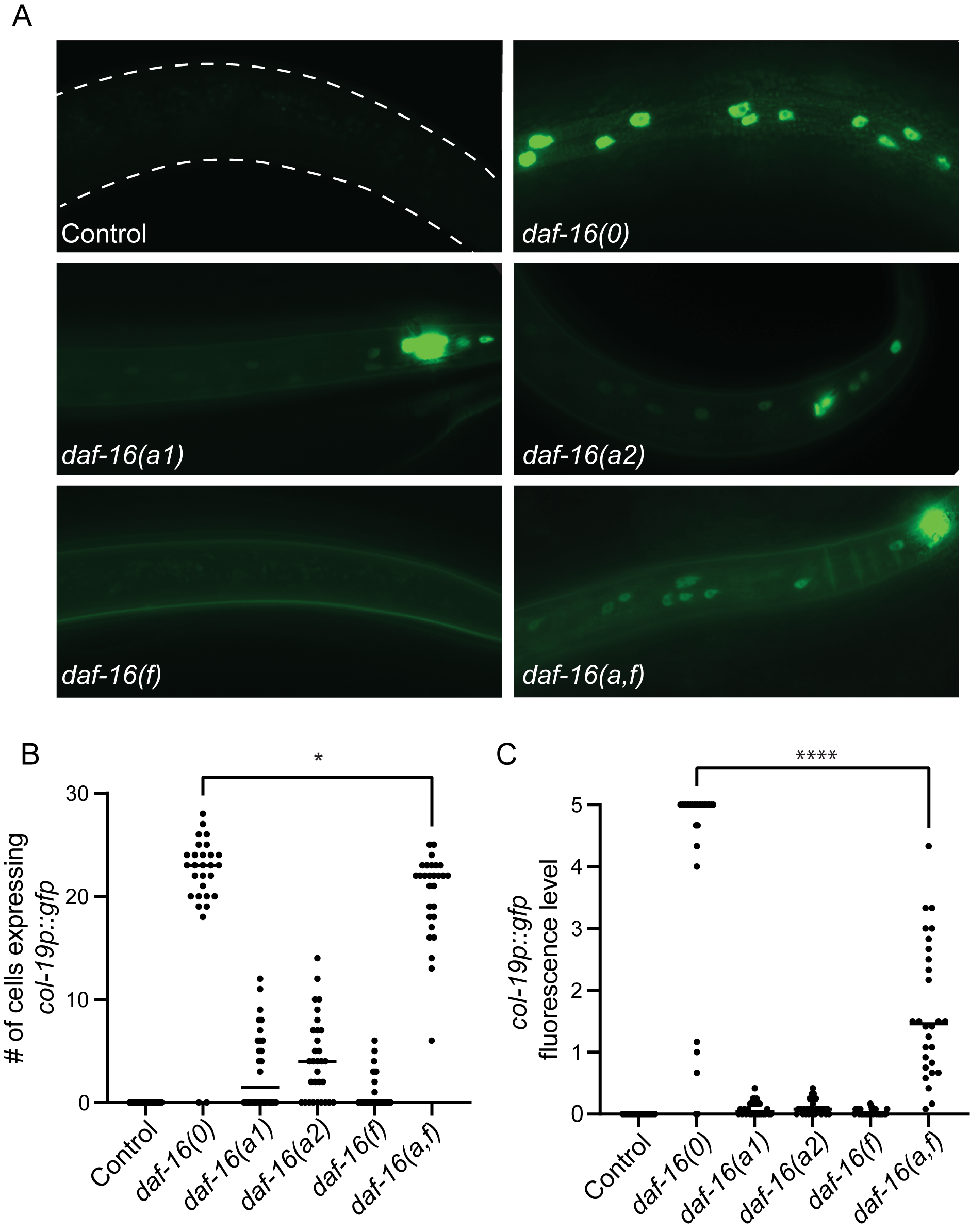

Supplement: S2 Fig — (A) Representative micrographs of dauer larvae scored in Fig 1D. In Fig 1D, any col-19p::gfp expression in the lateral hypodermis was considered “on”, however there are qualitative differences in that expression in the different strains. Dashed lines in the control indicate the boundaries of the dauer larva, as determined by DIC optics. (B) Loss of 1–2 isoforms of daf-16 results in fewer cells expressing col-19p::gfp compared to daf-16(0) dauer larvae. Quantification of the number of cells expressing col-19p::gfp along one side of the lateral hypodermis between the pharynx and rectum. Each dot represents a dauer larva. *p = 0.0460 (Two-tailed Mann-Whitney Test). (C) Loss of 1–2 isoforms of daf-16 results in dimmer expression of col-19p::gfp throughout the hypodermis compared to the daf-16(0) dauer larvae. Fluorescence levels were determined as described in S7 Fig. ****p<0.0001 (Two-tailed Mann-Whitney Test). Larvae shown in panels B-C were from an independent set of experiments from those in Fig 1D. n = 28–31; see S2 Table for complete underlying numerical data. (TIF) [file pgen.1009881.s002.tif]

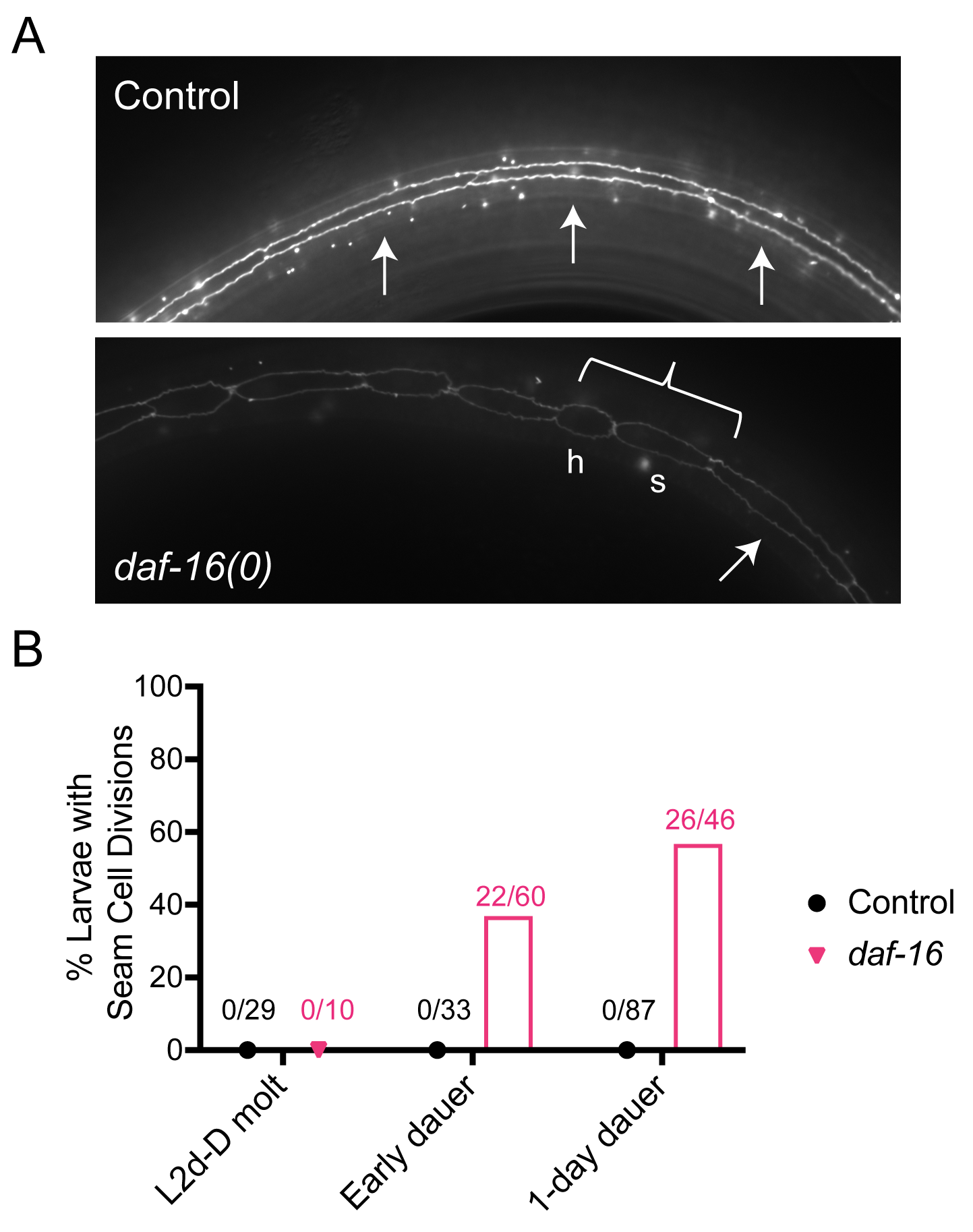

Supplement: S3 Fig — (A) Micrographs showing apical junctions between seam cells in daf-7 (control) and daf-16(0); daf-7 (daf-16(0)) dauer larva, and seam cell divisions in daf-16(0) dauer larvae. The worms are oriented with anterior to the left and ventral down. Apical junctions were visualized by ajm-1::gfp as part of the wIs78 transgene [42,78]. Junctions were present between all seam cells in both daf-7 control and daf-16(0); daf-7 dauer larvae. Several seam cells in the daf-16(0); daf-7 dauer shown have recently divided. An example of one dividing seam cell is bracketed. The region bounded by junctions is smaller and rounder in the anterior daughter that will fuse with hyp7 (h). The posterior daughter (s) will remain a seam cell. Seam cells that were not dividing are indicated with an arrow. These photos were taken soon after dauer formation (“early dauers”). (B) Percent of dauer larvae with one or more seam cells actively dividing at the time of scoring. Seam cell divisions were identified based on ajm-1::gfp expression. Cell divisions were only seen in daf-16(0) dauer larvae. (TIF) [file pgen.1009881.s003.tif]

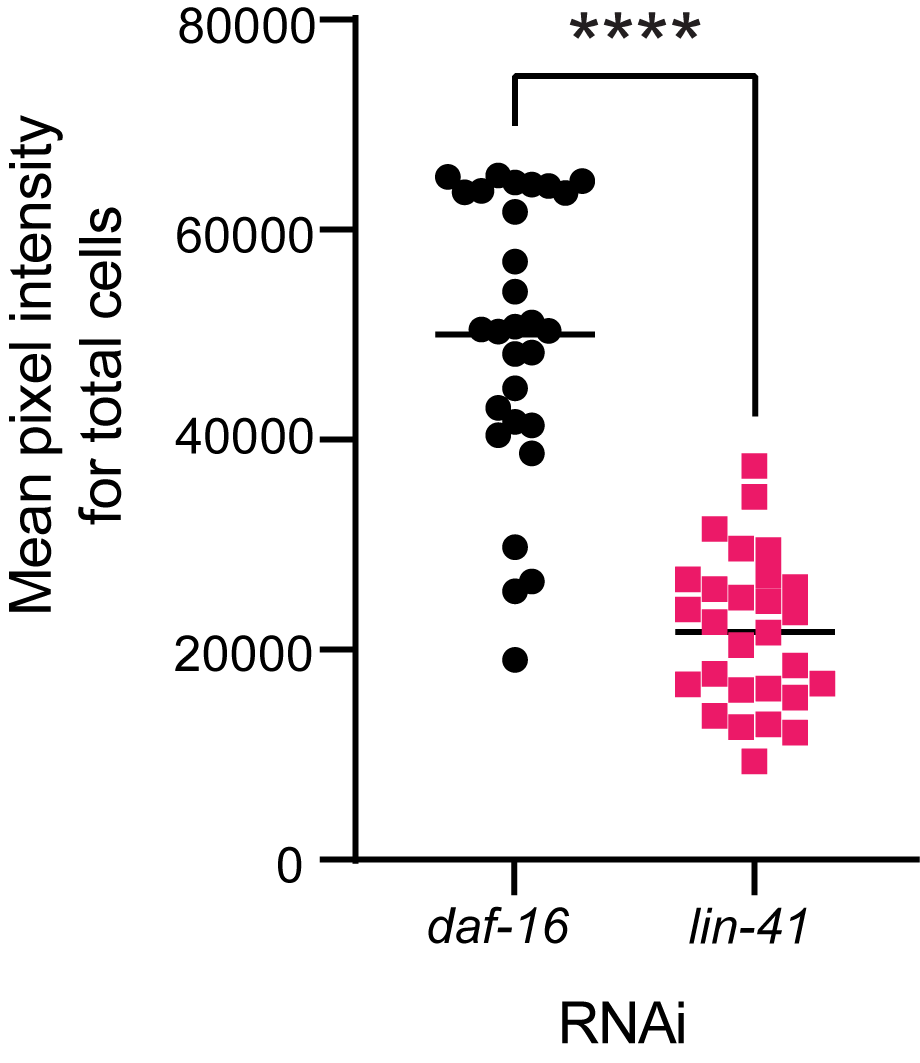

Supplement: S4 Fig — Larvae assessed for col-19p::gfp expression in Fig 2 were analyzed for fluorescence intensity using ImageJ. GFP-positive nuclei were outlined using the freehand tool and the mean pixel intensity for all of such nuclei were averaged together for each individual worm. These averages are underestimates for the expression of col-19p::gfp in daf-16(RNAi) dauer larvae because many nuclei had fully saturated pixels. ****p<0.0001 (Two-tailed Mann-Whitney Test). (n = 30). (TIF) [file pgen.1009881.s004.tif]

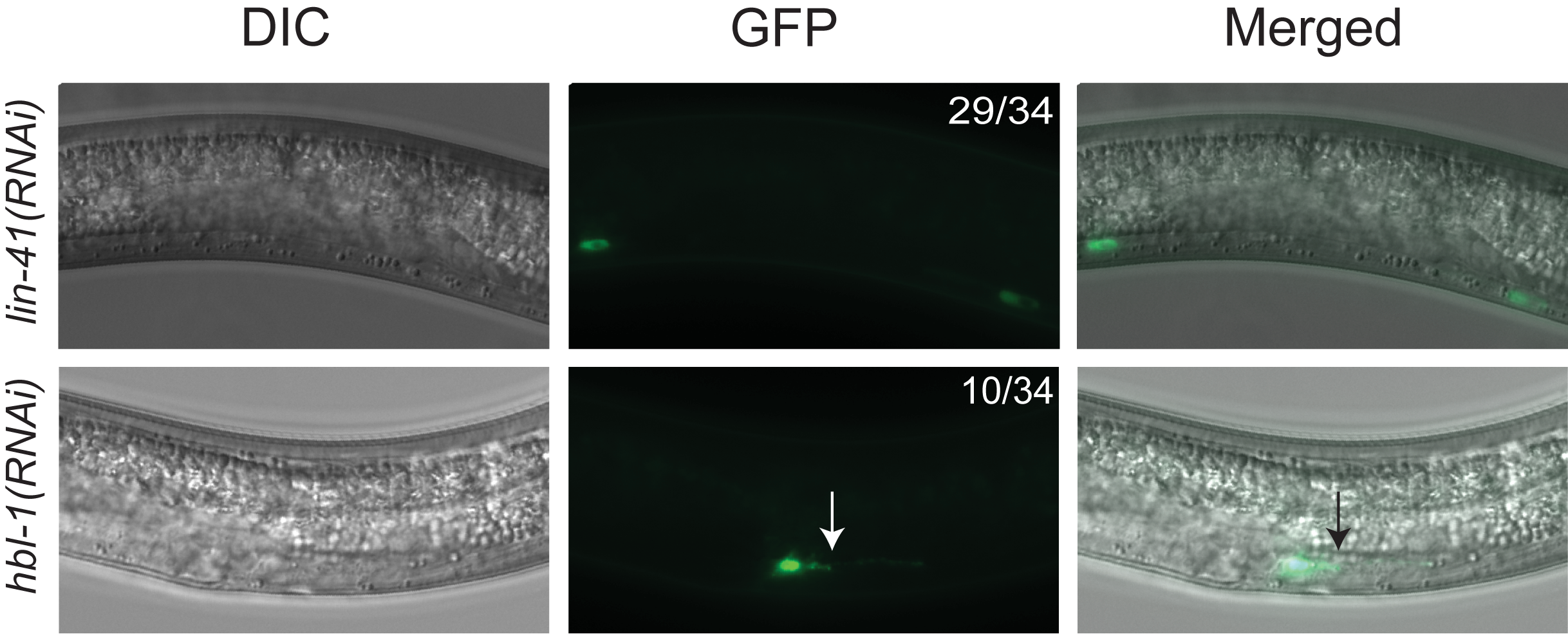

Supplement: S5 Fig — Numbers indicate larvae expressing col-19p::gfp in at least one cell in the relevant tissue over the total number of dauer larvae scored. Dauer larvae are oriented with anterior to the left and ventral down. (Top) lin-41 RNAi induces col-19p::gfp expression in vulva precursor cells (VPCs) during dauer at high penetrance. The larva shown expressed col-19p::gfp in P5.p and P7.p, but overall which VPCs expressed col-19p::gfp varied from worm to worm and did not correlate with the predicted cell fate they will adopt. The exposure time in the fluorescence channel was 200ms. (Bottom) hbl-1 RNAi induces col-19p::gfp expression in occasional ventral neurons during dauer at lower penetrance. As col-19p::gfp includes a nuclear localization sequence, expression is typically restricted to the nucleus. However, in the larva shown a neuronal process could be seen extending posteriorly to the cell body (arrow). The exposure time in the fluorescence channel was 50ms. (TIF) [file pgen.1009881.s005.tif]

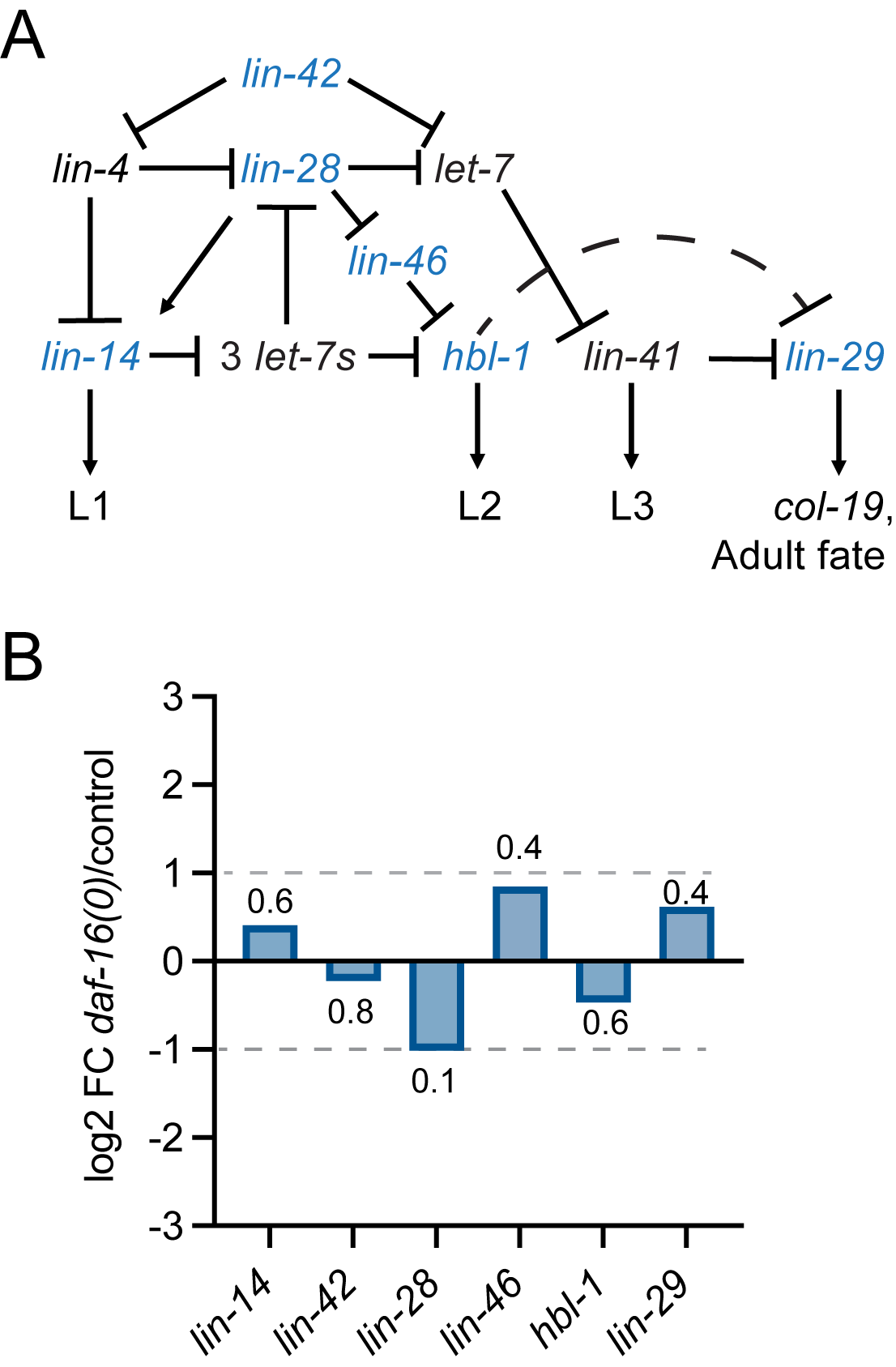

Supplement: S6 Fig — (A) A diagram of the network of heterochronic genes that regulates stage-specific seam cell fate during continuous development. “3 let-7s” indicates the let-7 family members, mir-48, mir-84, and mir-241. mir-48 is also downregulated by lin-42 (not depicted) [79]. All protein-coding genes except lin-41 are indicated in blue. (B) Log2 fold change of the protein-coding heterochronic genes other than lin-41, comparing daf-16(0) to control dauer larvae. Log2 fold change was calculated by DESeq2 from mRNA-seq data. Dashed lines indicate 2x fold upregulation or downregulation. False discovery rates (FDR) are indicated for each gene. (TIF) [file pgen.1009881.s006.tif]

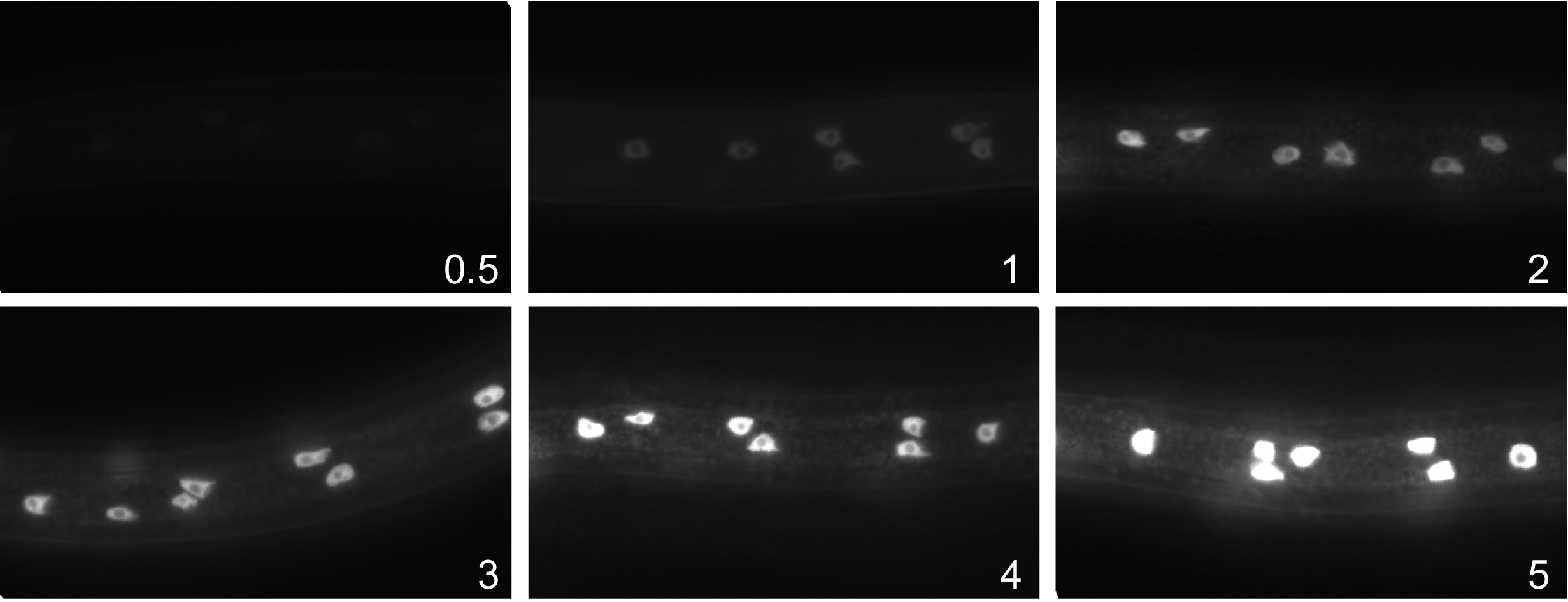

Supplement: S7 Fig — Fluorescence images of col-19p::gfp in dauer larvae were taken from a strain we found to produce a wide range of expression levels, XV160, daf-7(e1372); maIs105; unk-1(xk6). These images were taken using compound microscopy and identical settings, including an exposure time of 125ms. The images were then exported as 8-bit tiffs for ImageJ analysis. We identified six representative images distributed across the range of ImageJ values (0–250). We assigned numbers (0.5–5) to these images that reflect their distribution. A value of 0.25 was used if expression was visible, but less than that in the 0.5 panel. The fluorescence scale values with ImageJ values in parenthesizes were as follows: 0.5 (22), 1 (45), 2 (104), 3 (149), 4 (200), and 5 (250). These six images were then used as a scorecard to compare to experimental images for Figs 3B, 4A, and 5B. For these experiments, a 63x objective was used to take 2–3 images along the length of the dauer larva, using identical settings for all strains being compared. Each image was then subjectively compared to the fluorescence scale and assigned a value 0–5, with 0.5 values being used if an image was between two whole-number images. The values for each worm were averaged together to create an overall score for the worm. (TIF) [file pgen.1009881.s007.tif]

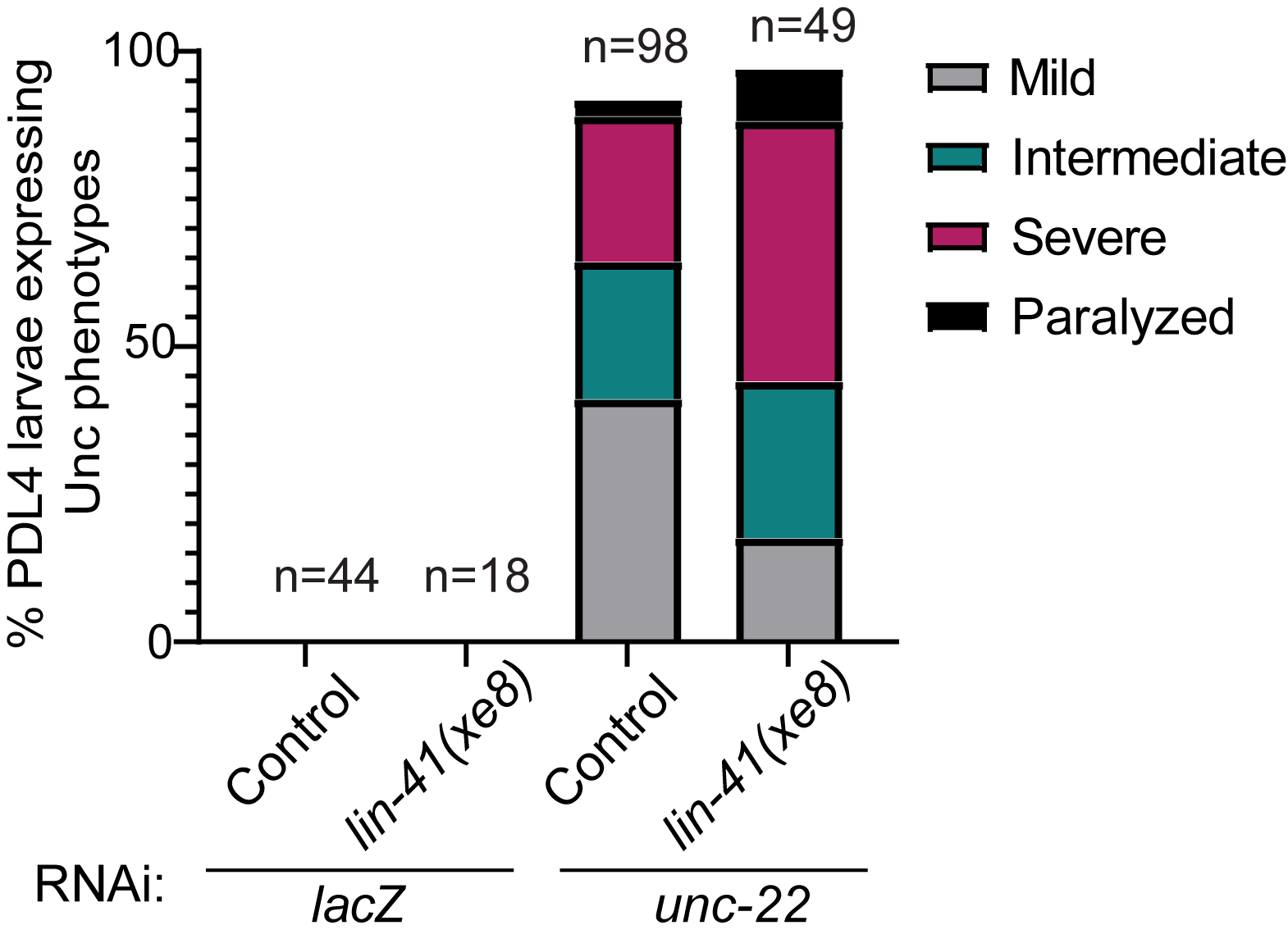

Supplement: S8 Fig — Unc phenotypes produced by unc-22(RNAi) were assessed during forward locomotion and binned into categories Mild: occasional twitching that did not interrupt movement. Intermediate: occasional twitching that did interrupt movement. Severe: constant twitching but still capable of forward locomotion. Paralyzed: worms whose twitching was so severe that they were not able to move forward. unc-22 RNAi experiments were carried out in parallel to the experiments shown in Fig 3B. However, because dauer larvae did not display strong Unc phenotypes, dauer larvae were moved to new RNAi plates at 20°C and allowed to recover to the post-dauer L4 (PDL4) stage. Numbers indicate the total number of PDL4 larvae assessed. (TIF) [file pgen.1009881.s008.tif]

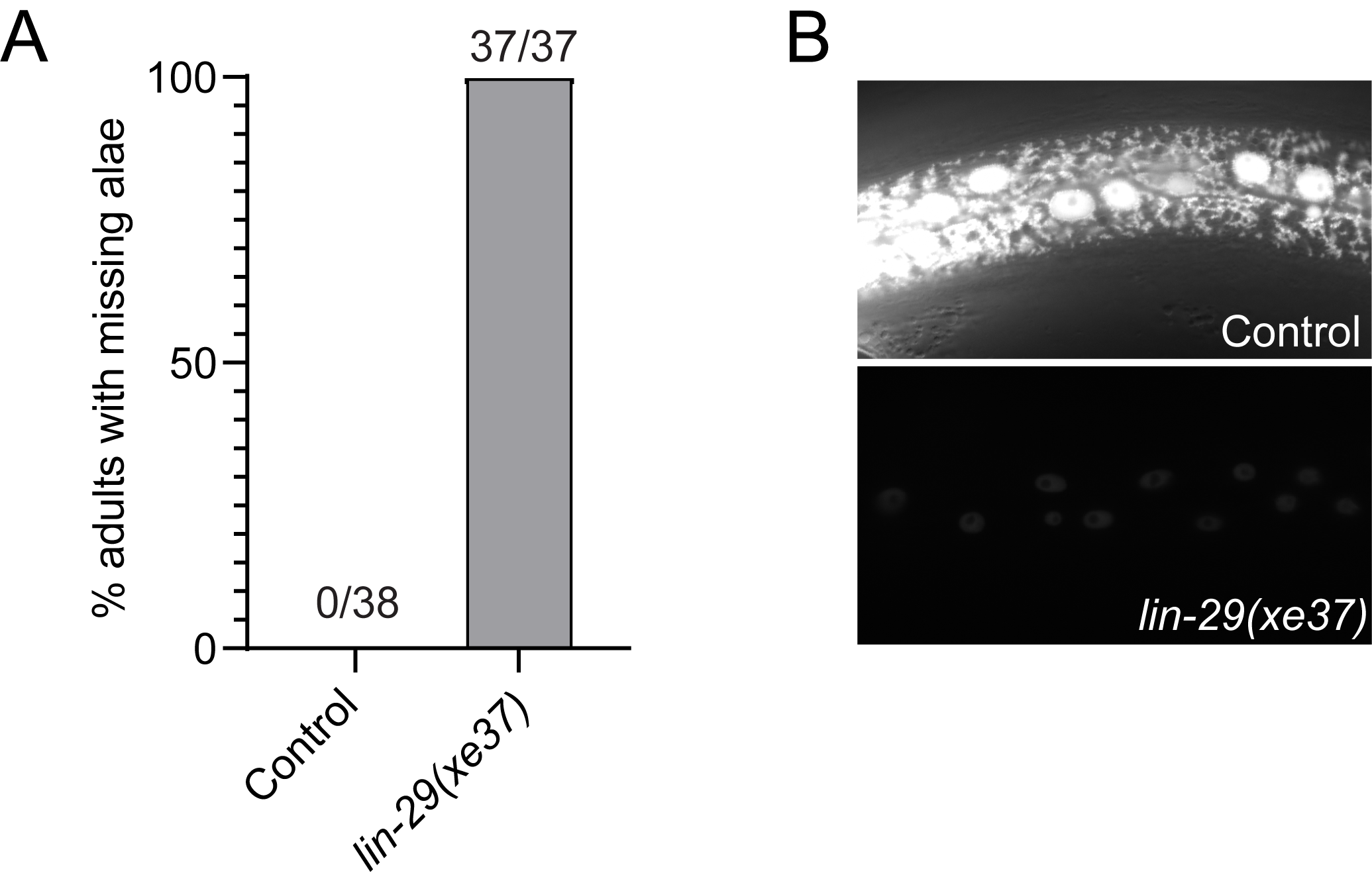

Supplement: S9 Fig — (A-B) The background for all strains was daf-7(e1372); maIs105[col-19p::gfp]. (A) lin-29(xe37) mutant adults do not have adult alae. Numbers indicate the number of worms with alae defects over the total number of adults. (B) lin-29(xe37) mutant adults express very low levels of col-19p::gfp. The two images were taken with identical settings (10ms exposure). Representative images are shown (n = 37–38). (TIF) [file pgen.1009881.s009.tif]

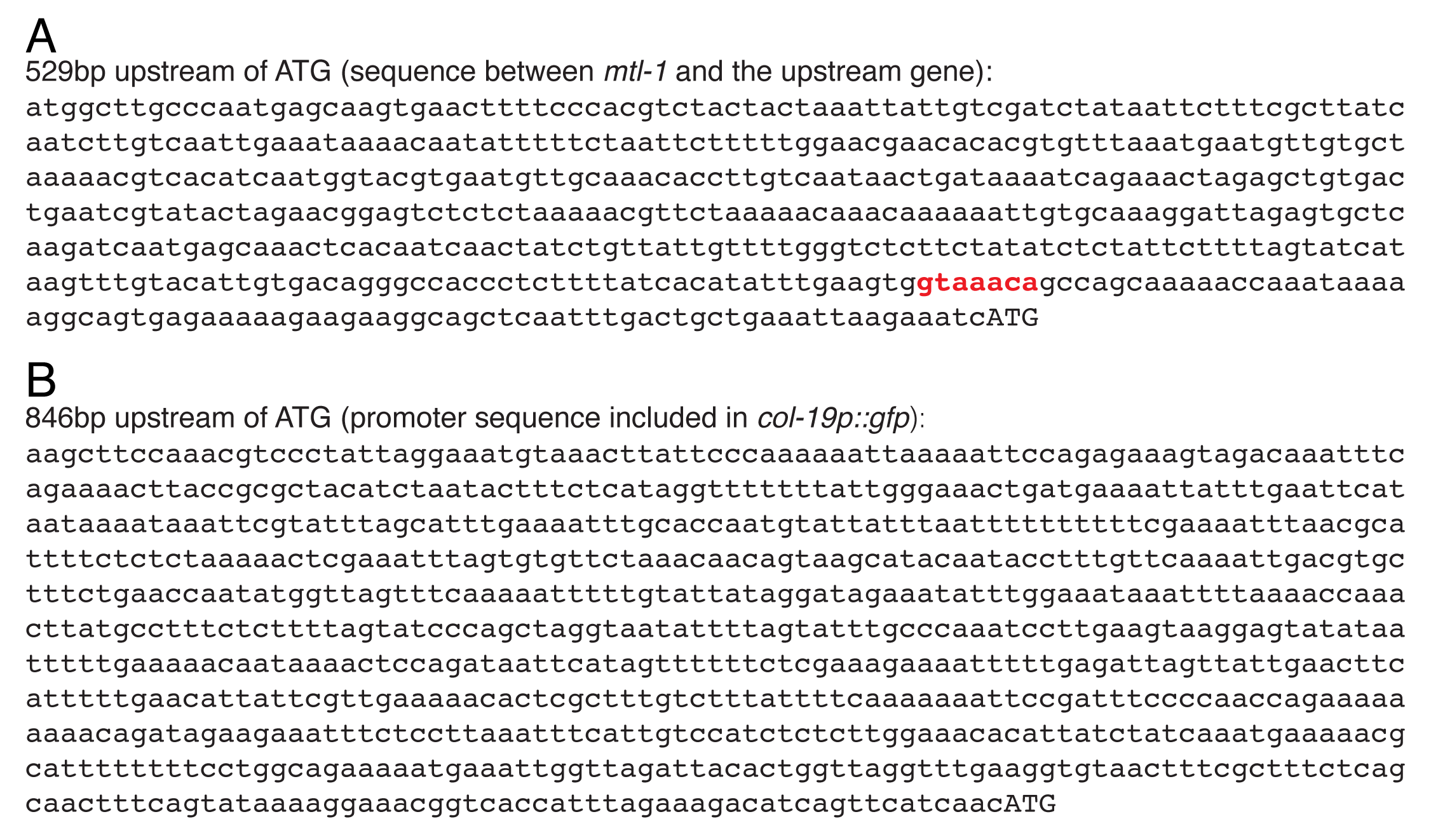

Supplement: S10 Fig — (A) Genetic region upstream of mtl-1, a confirmed transcriptional target of DAF-16 [52,53]. DNA sequence beginning immediately 3’ to W02F12.8, the gene upstream of mtl-1 is shown. This sequence likely contains promoter elements that regulate mtl-1 expression. A canonical DAF-16-Binding Element (DBE, red) is located 76bp upstream of the mtl-1 ATG. (B) The 846bp upstream of the col-19 ATG which comprises the regulatory sequence driving col-19p::gfp expression [28]. No canonical DBEs (GTAAACA or TGTTTAC) [49] were found in this sequence. Sequences were taken from WormBase (WS280). (TIF) [file pgen.1009881.s010.tif]

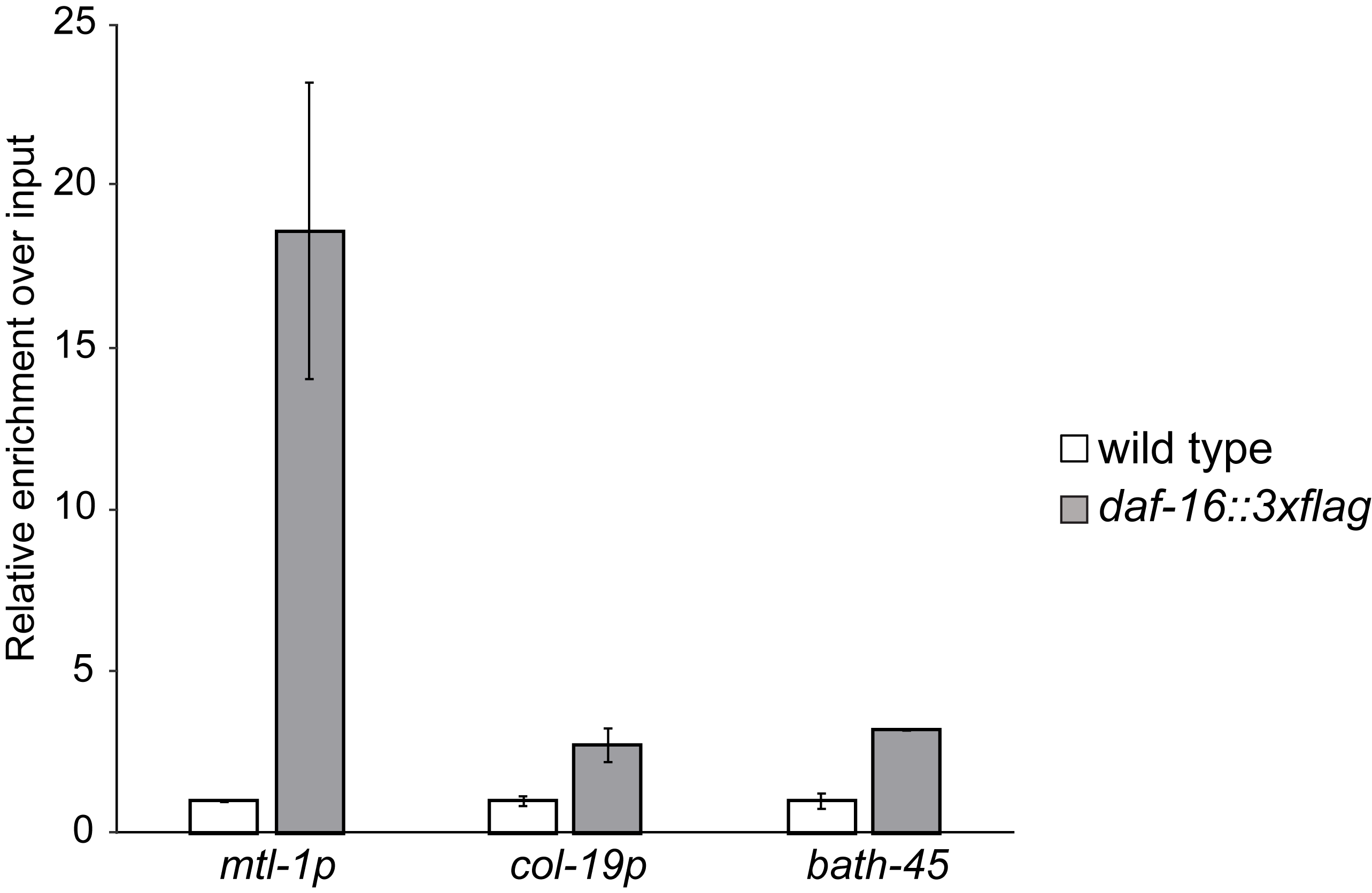

Supplement: S11 Fig — ChIP-qPCR experiments were performed on N2 or daf-16(ar620[daf-16::zf1-wrmScarlet-3xFLAG]) dauer larvae. Binding of DAF-16-3xFLAG was first normalized to input, and then to the average of the respective wild-type value (mean +/- SD for two technical replicates) is shown. Binding to the col-19 promoter was not observed, whereas there was substantial binding to the promoter of the known DAF-16 target mtl-1 (Barsyte et al. 2001; Li et al. 2008). The coding region of the heterochromatinized gene bath-45 was used as negative control. This figure shows the second of two biological replicates; the first replicate is shown in Fig 6A. (TIF) [file pgen.1009881.s011.tif]

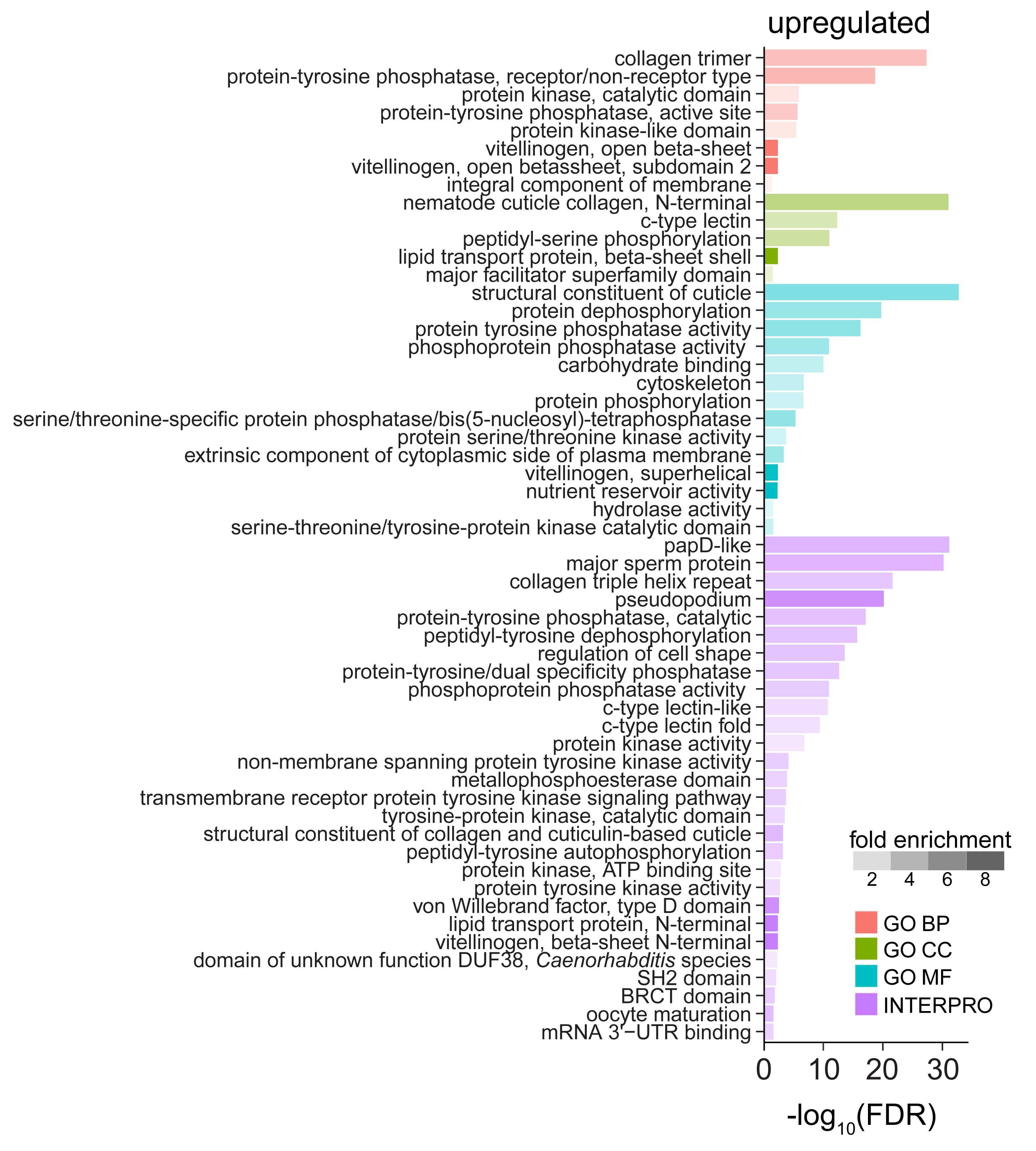

Supplement: S12 Fig — DAVID analysis showing gene ontology (GO) and InterPro terms that were significantly enriched (p ≤ 0.05, Bonferroni corrected) in genes whose expression was upregulated ≥2x (FDR ≤ 0.05) in daf-16(0); daf-7 dauer larvae vs. daf-7 (control) dauer larvae. GO BP = GO term biological process; GO CC = GO term cellular compartment; GO MF = GO term molecular function, INTERPRO = InterPro protein classification. (TIF) [file pgen.1009881.s012.tif]

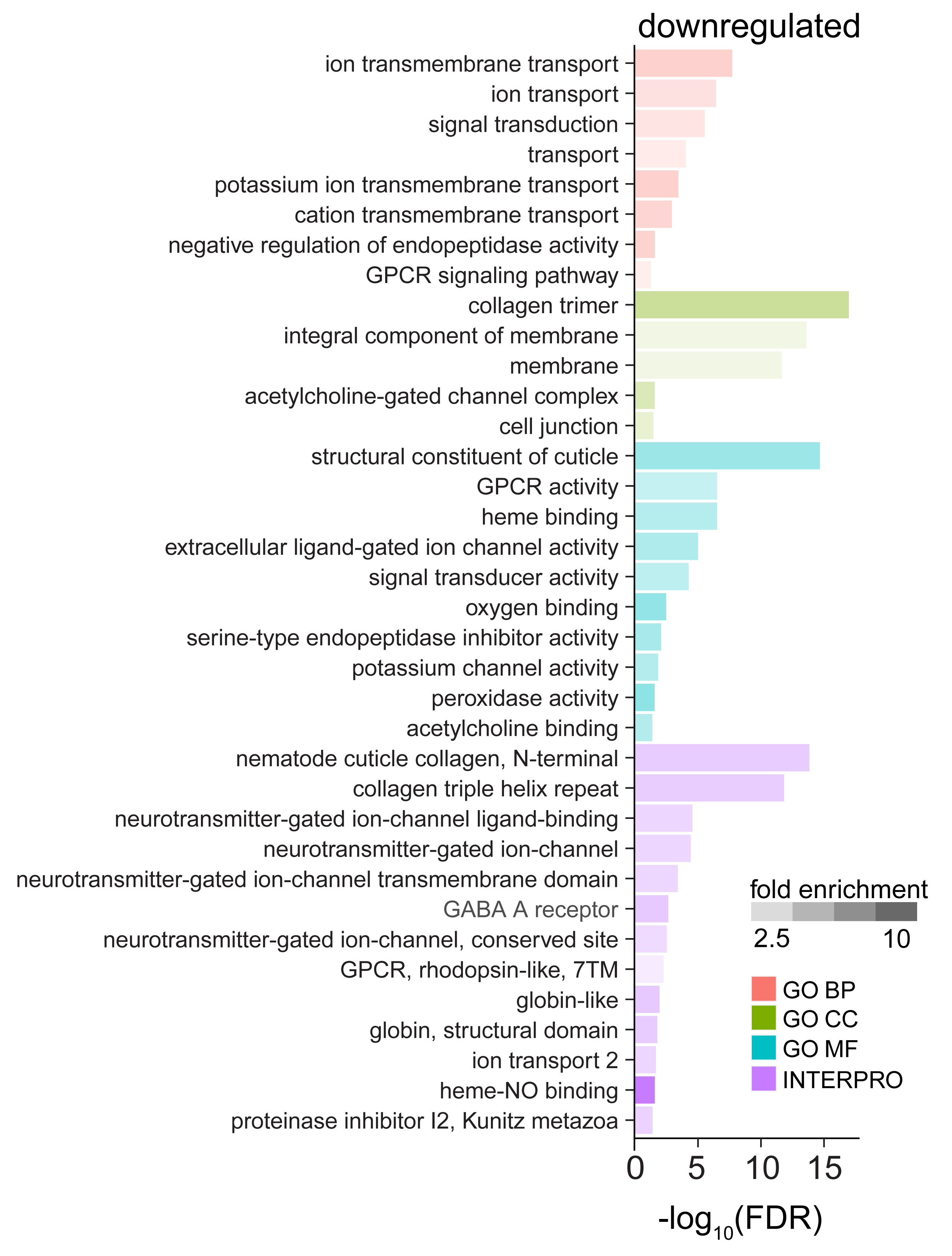

Supplement: S13 Fig — DAVID analysis showing gene ontology (GO) and InterPro terms that were significantly enriched (p ≤ 0.05, Bonferroni corrected) in genes whose expression was downregulated ≥2x (FDR ≤ 0.05) in daf-16(0); daf-7 dauer larvae vs. daf-7 (control) dauer larvae. GO BP = GO term biological process; GO CC = GO term cellular compartment; GO MF = GO term molecular function, INTERPRO = InterPro protein classification. (TIF) [file pgen.1009881.s013.tif]

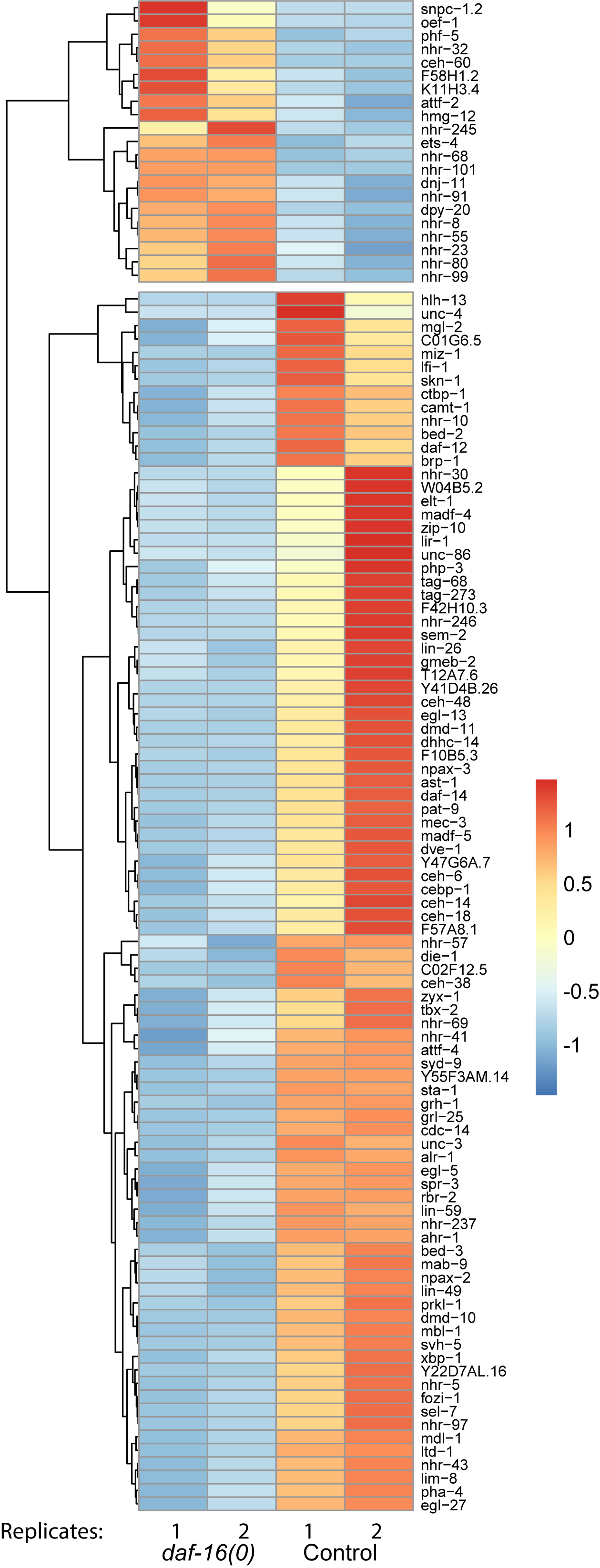

Supplement: S14 Fig — Genes encoding transcription factors were defined based on their WormBase annotation (WS279). Although annotated as transcription factors, we noticed that the WormBase list also contained genes encoding RNA-binding proteins [80,81]. We therefore subtracted genes identified as encoding RNA-binding proteins from the transcription factor list. From among these genes, we searched our mRNA-seq data to find those whose expression changed ≥2x (FDR ≤ 0.05) by DESeq2 analysis. This analysis produced 112 genes that fit our criteria. Those genes are shown here as heat maps depicting relative expression of these genes in daf-16(0); daf-7 dauer larvae or daf-7 (control) dauer larvae from mRNA-seq data. The row-normalized reads for each of two biological replicates per strain are depicted. (TIF) [file pgen.1009881.s014.tif]
