# Supplementary material for: daf-16/FOXO blocks adult cell fate in Caenorhabditis elegans dauer larvae via lin-41/TRIM71
Source: PLoS Genet. 2021 Nov 15;17(11):e1009881. doi: 10.1371/journal.pgen.1009881 (PMC8629381; doi:10.1371/journal.pgen.1009881)
Supplement: S1 Table — (DOCX) [file pgen.1009881.s015.docx]

**Table 1. List of strains used in this study.**

| **Figure** | **Strain** | **Genotype** | **References** |
| --- | --- | --- | --- |
| Fig 1A | VT1777 | *daf-7(e1372); maIs105[col-19p::gfp]* | *maIs105:* [1] |
|  | XV36 | *daf-16(mgDf50); daf-7(e1372); maIs105* | *mgDf50:* [2] |
|  | XV156 | *daf-16(mu86); daf-7(e1372); maIs105* | *mu86:* [3] |
| Fig 1B | VT1777 | *daf-7(e1372); maIs105* |  |
|  | XV36 | *daf-16(mgDf50); daf-7(e1372); maIs105* |  |
| Fig1C | XV33 | *maIs105* |  |
|  | VT1750 | *daf-16(mgDf50); maIs105* |  |
| Fig 1D | VT1777 | *daf-7(e1372); maIs105* |  |
|  | XV36 | *daf-16(mgDf50); daf-7(e1372); maIs105 (= daf-16(0))* |  |
|  | XV72 | *daf-16(tm5030); daf-7(e1372); maIs105 (= daf-16(a1))* | *tm5030, tm5032, tm6659:* [4] |
|  | XV73 | *daf-16(tm5032); daf-7(e1372); maIs105 (= daf-16(a2))* |  |
|  | XV71 | *daf-16(tm6659); daf-7(e1372); maIs105 (= daf-16(f))* |  |
|  | XV74 | *daf-16(mg54); daf-7(e1372); maIs105 (= daf-16(a,f))* | *mg54:* [2] |
| Fig 1E | CB1372 | *daf-7(e1372)* |  |
|  | VT2317 | *daf-16(mgDf50); daf-7(e1372)* |  |
| Fig 2B | VT1777 | *daf-7(e1372); maIs105* |  |
| Fig 3A | CB1372 | *daf-7(e1372)* |  |
|  | VT2317 | *daf-16(mgDf50); daf-7(e1372)* |  |
| Fig 3B | VT1777 | *daf-7(e1372); maIs105[col-19p::gfp]* |  |
|  | XV181^1^ | *lin-41(xe8[∆3’UTR])/lin-41(bch28[Peft3::gfp::h2b::tbb-2 3’UTR] xe70[∆3’UTR]); daf-7(e1372); maIs105* | *xe8:* [5]*. bch28 xe70:* [6] |
| Fig 4A | VT1777 | *daf-7(e1372); maIs105[col-19p::gfp]* |  |
|  | XV253 | *lin-29(xe37); daf-7(e1372); maIs105* | *xe37:* [6] |
| Fig 4B | VT1777 | *daf-7(e1372); maIs105* |  |
|  | XV238^2^ | *lin-41(n2914)/nIs408[lin-29::mCherry, ttx-3p::gfp]; daf-7(e1372); maIs105* | *n2914:* [7]  *nIs408:* [8] |
|  | XV245 | *nIs408; daf-7(e1372); maIs105* |  |
|  | XV239^2^ | *lin-41(n2914)/nIs408; lin-29(n546); daf-7(e1372); maIs105* |  |
| Fig 4C | HW1822 | *lin-29(xe61[lin-29::gfp::3xflag])* (for L3 experiments) | *xe61:* [9] |
|  | XV243 | *lin-29(xe61); daf-7(e1372)* (for dauer experiments) |  |
| Fig 4D | XV243 | *lin-29(xe61); daf-7(e1372)* |  |
| Fig 5A | XV36 | *daf-16(mgDf50); daf-7(e1372); maIs105* |  |
|  | XV87 | *daf-16(mgDf50); lin-29(n546); daf-7(e1372); maIs105* |  |
|  | XV254 | *daf-16(mgDf50); lin-29(xe37); daf-7(e1372); maIs105* |  |
| Fig 5B | VT1777 | *daf-7(e1372); maIs105* |  |
|  | XV36 | *daf-16(mgDf50); daf-7(e1372); maIs105* |  |
|  | XV254 | *daf-16(mgDf50); lin-29(xe37); daf-7(e1372); maIs105* |  |
| Fig 6A | N2 | wild-type |  |
|  | GS8924 | *daf-16(ar620[daf-16::zf1-wrmScarlet-3xFLAG])* | Gift from K. Luo, Greenwald lab |
| Fig 6B | CB1372 | *daf-7(e1372)* |  |
|  | VT2317 | *daf-16(mgDf50); daf-7(e1372)* |  |
| Fig S1A | N2 | *wild type* |  |
|  | VT1777 | *daf-7(e1372); maIs105* |  |
|  | XV138 | *daf-2(e1370); maIs105* |  |
| Fig S1B | XV138 | *daf-2(e1370); maIs105* |  |
|  | XV115 | *daf-5(m512); daf-2(e1370); maIs105* | *m512:* [10] |
| Fig S2 | VT1777 | *daf-7(e1372); maIs105* |  |
|  | XV36 | *daf-16(mgDf50); daf-7(e1372); maIs105* |  |
| Fig S2 | XV72 | *daf-16(tm3050); daf-7(e1372); maIs105 (= daf-16(a1))* |  |
|  | XV73 | *daf-16(tm3052); daf-7(e1372); maIs105 (= daf-16(a2))* |  |
|  | XV71 | *daf-16(tm6659); daf-7(e1372); maIs105 (= daf-16(f))* |  |
|  | XV74 | *daf-16(mg54); daf-7(e1372); maIs105 (= daf-16(a,f))* |  |
| Fig S3 | XV27 | *daf-7(e1372); wIs78[scm::gfp, ajm-1::gfp]* | *wIs78:* [11,12] |
|  | XV29 | *daf-16(mgDf50); daf-7(e1372); wIs78[scm::gfp, ajm-1::gfp]* |  |
| Fig S4 | VT1777 | *daf-7(e1372); maIs105* |  |
| Fig S5 | VT1777 | *daf-7(e1372); maIs105* |  |
| Fig S6 | CB1372 | *daf-7(e1372)* |  |
|  | VT2317 | *daf-16(mgDf50); daf-7(e1372)* |  |
| Fig S7 | XV160 | *daf-7(e1372); maIs105; unk-1(xk6)* |  |
| Fig S8 | VT1777 | *daf-7(e1372); maIs105* |  |
|  | XV181 | *lin-41(xe8)/lin-41(bch28 xe70); daf-7(e1372); maIs105* |  |
| Fig S9 | VT1777 | *daf-7(e1372); maIs105* |  |
|  | XV253 | *lin-29(xe37); daf-7(e1372); maIs105* |  |
| Fig S11 | N2 | wild-type |  |
|  | GS8924 | *daf-16(ar620[daf-16::zf1-wrmScarlet-3xFLAG])* |  |

1. Strain XV181 was maintained as heterozygotes. The relevant homozygous progeny were isolated the day before the experiment. Homozygous *xe8* progeny were recognizable by lack of GFP expression from *bch28.*
2. Strains XV238 and XV239 were maintained as heterozygotes. For experiments individual gravid hermaphrodites were cloned out to several 60 mm plates and allowed to lay embryos for 3-5 hours at 24˚C. Parents were then removed. Plates where 100% of larvae expressed *ttx-3p::gfp* were discarded. From the remaining plates, all dauer larvae were scored for both *ttx-3p::gfp* expression (indicating *nIs408)* and *col-19p::gfp* expression.

**References**

1. Feinbaum R, Ambros V. The timing of *lin-4* RNA accumulation controls the timing of postembryonic developmental events in *Caenorhabditis elegans*. Developmental Biology. 1999;210: 87–95. doi:10.1006/dbio.1999.9272

2. Ogg S, Paradis S, Gottlieb S, Patterson GI, Lee L, Tissenbaum HA, et al. The Fork head transcription factor DAF-16 transduces insulin-like metabolic and longevity signals in *C. elegans.* Nature. 1997;389: 994–999. doi:10.1038/40194

3. Lin K, Dorman JB, Rodan A, Kenyon C. *daf-16:* An HNF-3/forkhead family member that can function to double the life-span of *Caenorhabditis elegans*. Science. 1997;278: 1319–1322. doi:10.1126/science.278.5341.1319

4. Chen AT-Y, Guo C, Itani OA, Budaitis BG, Williams TW, Hopkins CE, et al. Longevity genes revealed by integrative analysis of isoform-specific *daf-16/*FoxO mutants of *Caenorhabditis elegans*. Genetics. 2015;201: 613–629. doi:10.1534/genetics.115.177998

5. Ecsedi M, Rausch M, Großhans H. The *let-7* microRNA directs vulval development through a single target. Developmental Cell. 2015;32: 335–344. doi:10.1016/j.devcel.2014.12.018

6. Aeschimann F, Neagu A, Rausch M, Großhans H. *let-7* coordinates the transition to adulthood through a single primary and four secondary targets. Life Sci Alliance. 2019;2: e201900335. doi:10.26508/lsa.201900335

7. Slack FJ, Basson M, Liu Z, Ambros V, Horvitz HR, Ruvkun G. The *lin-41* RBCC gene acts in the *C. elegans* heterochronic pathway between the *let-7* regulatory RNA and the LIN-29 transcription factor. Molecular Cell. 2000;5: 659–669.

8. Harris DT, Horvitz HR. MAB-10/NAB acts with LIN-29/EGR to regulate terminal differentiation and the transition from larva to adult in *C. elegans*. Development. 2011;138: 4051–4062. doi:10.1242/dev.065417

9. Aeschimann F, Kumari P, Bartake H, Gaidatzis D, Xu L, Ciosk R, et al. LIN41 Post-transcriptionally silences mRNAs by two distinct and position-dependent mechanisms. Mol Cell. 2017;65: 476-489.e4. doi:10.1016/j.molcel.2016.12.010

10. Tewari M, Hu PJ, Ahn JS, Ayivi-Guedehoussou N, Vidalain P-O, Li S, et al. Systematic interactome mapping and genetic perturbation analysis of a *C. elegans* TGF-β signaling network. Mol Cell. 2004;13: 469–482. doi:10.1016/s1097-2765(04)00033-4

11. Koh K, Rothman JH. ELT-5 and ELT-6 are required continuously to regulate epidermal seam cell differentiation and cell fusion in *C. elegans*. Development. 2001;128: 2867–2880.

12. Abrahante JE, Daul AL, Li M, Volk ML, Tennessen JM, Miller EA, et al. The *Caenorhabditis elegans* *hunchback*-like gene *lin-57/hbl-1* controls developmental time and is regulated by microRNAs. Developmental Cell. 2003;4: 625–637.
