# Supplementary material for: daf-16/FOXO blocks adult cell fate in Caenorhabditis elegans dauer larvae via lin-41/TRIM71
Source: PLoS Genet. 2021 Nov 15;17(11):e1009881. doi: 10.1371/journal.pgen.1009881 (PMC8629381; doi:10.1371/journal.pgen.1009881)
Supplement: Attachment — Submitted filename: Response to Reviewers.docx [file pgen.1009881.s024.docx]

We thank the reviewers for their enthusiasm for our work and for their insightful comments, which have improved the clarity of our manuscript. Below are our responses to their specific points.

**Reviewer 1**

1. I have just one minor critique: The pathway model shown in Figure 2A shows a positive role for lin-14 in promoting lin-28 activity and shows the let-7 family ‘sister’ microRNAs as regulating only hbl-1. However, an alternative depiction of the network would place the let-7 family microRNAs downstream of lin-14 (which appears to regulate their transcription (Tsialikas et al (2017) Genetics 205: 251-262)), and upstream of both lin-28 and hbl-1.

**We thank the reviewer for this suggestion. We agree and have revised the figure accordingly. In addition, we have incorporated the information from Azzi et al. (2020) PMID: 32223899, demonstrating that *hbl-1* also regulates *lin-29* expression. We have similarly modified the model in figure S6 (formerly figure S5).**

**Reviewer 2**

1. Please include line numbers.

**Done.**

2. Introduction, second paragraph, second-to-last line: delete the Pierce et al. reference.

**Done.**

3. Throughout the manuscript, "DAF-16/FOXO" should be used to improve clarity for readers not familiar with C. elegans.

**Done.**

4. Introduction, second paragraph, last line: the two references listed address the regulation of DAF-16 subcellular localization, not target genes. I would consider citing a dauer review (e.g., Fielenbach and Antebi).

**We agree that those references dealt with only the part of the sentence describing regulation of DAF-16 activity and not target genes and that review articles could better encompass all of the points. We replaced the original references with Fielenbach and Antebi (2008) and Murphy and Hu (2013).**

5. Results, first paragraph line 12 and second paragraph lines 3-4: Stating that daf-16 and daf-5 mutant larvae are "dauer-defective" could be confusing to non-mavens. I would consider being more specific to enhance clarity, e.g., "As larvae with reduced daf-16 activity bypass dauer arrest in the context of reduced DAF-2 insulin signaling,..." and an analogous sentence for daf-5.

**We have altered these sentences to say “As *daf-16(-)* larvae are normally unable to enter dauer (i.e. dauer-defective)…” and “Similar to *daf-16(-)* larvae, *daf-5(0)* larvae do not normally develop through dauer.”.**

6. Figures 1D and S2: Since the col-19p::GFP expression phenotypes of daf-16(0) and daf-16a/f mutants do not differ in terms of penetrance (1D) but do appear to differ in terms of GFP expression levels per cell and number of cells/animal, both expression levels and number of GFP+ cells in these two mutant backgrounds should be quantified.

**These data have been added to figure S2.**

7. Paragraph entitled “daf-16 promotes expression of lin-41 during dauer,” lines 10-11: Similar to 6., GFP expression in lin-41(RNAi) and daf-16(RNAi) should be quantified.

**These data have been added in a new supplemental figure, S4.**

8. Figure 1E: what do "1" and "2" denote?

**These numbers indicate replicates. This terminology has been clarified in the figure.**

9. Figure 2, legend: consider changing “sisters” to “family members.”

**Done.**

10. Figure 2B: it would be nice to include a daf-16 RNAi sample in this experiment so that the relative effects of daf-16 RNAi and lin-41 RNAi on col-19p::GFP expression could be visualized (differences in RNAi efficiency notwithstanding).

**We have added an image of the *daf-16* RNAi controls that we performed to this figure.**

11. Figure S1, legend, line 4: "da Graca et al."

**Corrected.**

12. Figure S7: typo: "paralyzed"

**Corrected.**

13. Figure S8B: I don't think the 250ms exposure adds anything.

**We agree and have removed those panels.**

14. Table 1: allele names for the daf-16a mutants are "tm5030" and "tm5032."

**Corrected.**

**Reviewer 3**: 

I have several questions of things that were unclear to me and should be clairified:

1. Are these adult-specific collagens expressed in seam cells or hypodermis? If not in seam cells, how does this relate to loss of multipotency?

**This is an excellent point. By far the best studied gene encoding an adult-enriched collagen is *col-19. col-19* expression has been studied primarily using transcriptional reporters, including the *col-19p::gfp* used in this study. These *col-19* reporters are expressed in both seam cells and hyp7 (and other hypodermal cells) in adults. Interestingly, the adult-specific timing of expression applies to all of these cell types, even though hyp7 would be considered differentiated at all stages. Therefore, while the multipotency vs. differentiation question only applies to seam cells, larval vs. adult cell fate applies in some way to all hypodermal cells. While the study of heterochronic genes often focuses on seam cells, heterochronic mutants cause changes in expression of *col-19* reporters in both seam cells and hyp7, though sometimes one cell-type is affected more strongly than the other.**

**The only other adult collagen-encoding gene whose expression in seam cells vs. hyp7 has been studied using a reporter is *col-7,* which is also expressed in both seam and hyp7 in adults (Liu et al., 1995, PMID 2628162). However, data from a genome-wide study using poly-A-binding protein expressed tissue-specifically to pull down mRNAs suggests that most other adult-enriched collagens are also expressed in both seam cells and hyp7 (Blazie et al., 2017, PMID: 28348061).**

**We have added a supplemental table (S3) summarizing the data about collagen expression in seam vs. hyp7 and its significance. In the main text, we have added this sentence: “Similar to col-19p::gfp, most of these adult-enriched collagens appear to be expressed both in seam cells and hyp7 (Table S3)”.**

2. Is col-19 also misexpressed in daf-16(null) dauers induced by starvation? Or is this specific to the tgf beta pathway?

**This is another excellent question that we would love to address. Unfortunately, *daf-16(null)* larvae do not enter dauer in response to starvation. Even *daf-16; daf-7* strains that do enter dauer at 25˚C do not form dauers on starved plates. We have also been unable to induce dauer formation in *daf-16(null)* dauer larvae using dauer pheromone when *daf-7* is wild-type.**

**We have clarified this point in lines 124–126 where we say “As *daf-16(-)* larvae are normally unable to enter dauer even in dauer-inducing environmental conditions, we took advantage of the temperature-sensitive, dauer-constitutive allele, *daf-7(e1372)*, to enable dauer formation in *daf-16(-)* larvae”.**

Minor issues:

1. Manuscript could be more concise and less repetitive across sections (intro, results, discussion)

**We have gone through the manuscript to remove unnecessary repetition while retaining enough information to make each section understandable on its own.**

2. Compared to PMID: 9649524, how do the findings add conceptional novel insights?.

**In lines 376–380, we state “For example, many heterochronic genes that are essential for stage-specific cell fate specification during continuous development are dispensable after dauer (Liu and Ambros 1991; Abrahante et al. 1998, 2003; Karp and Ambros 2012). However, the mechanisms that act to prevent precocious specification within the dauer stage itself have not been addressed.” (Emphasis added here).**

**The paper mentioned above described the results of a genetic screen that took advantage of *col-19p::gfp* to identify novel heterochronic genes that function downstream of *lin-4* during continuous development. The authors provided an important contribution to the field, including notably new alleles and characterization of *lin-42* and the discovery of *mir-48 (“lin-58”* in the paper)*.* However, that paper did not examine the regulation of cell fate during dauer. The only connection to dauer in their paper is that they checked to see whether the phenotypes of their newly identified mutants were suppressed by passage through dauer, which is the context in which we cite their paper in line 378. Our work did not describe a genetic screen and instead is focused on the regulation of adult cell fate during the dauer stage and the role of *daf-16* in this context. Therefore, we do not see overlap between their study and our work, other than the broad study of heterochronic genes and the use of *col-19p::gfp* as a tool.**

Also other genes like lin-42, lin-28, mir-41 etc that are upstream of lin-29 and regulate Pcol-19::GFP were not discussed or investigated

**With respect to upstream genes in the heterochronic pathway, we do provide some insight by showing that RNAi of *lin-14* and *hbl-1* produces a minimal effect on *col-19p::gfp* during dauer (Fig 2) and that mRNA levels of the other major protein-coding heterochronic genes are unaffected in *daf-16(0)* dauer larvae (Fig S6, formerly Fig S5). However, *lin-42* was missing from this figure, so we have added that information. *lin-42* expression is also unaffected in *daf-16(0)* dauer larvae. The question of how *daf-16* regulates *lin-41* and whether that regulation occurs via microRNAs such as *let-7* is one we broach in the discussion and is an area of active research.**

3. The transcription factor lin-29 is not required. The authors mention 112 transcription factors from their RNA seq and suggest that they in the absense of daf-16 regulate misexpression of adult collagens or loss of mulitpotency. This could be easily tested by a small RNAi screen using Pcol-19::GFP as a read out. I am not saying they need to do this, but it would be nice to get some insight of a potential mechanism here.

**We agree with this reviewer that this screen is an important next step. We decided not to try to do it as part of this paper for two reasons. First, we think that there are enough significant new insights in our story in its current form to justify publication in PLoS Genetics. Second, we want to take the time to do the screen carefully and validate the results before publishing. We are excited to see what we find.**
